# Macrophages Inhibit Ciliary Protein Levels by Secreting BMP-2 Leading to Airway Epithelial Remodeling Under Cigarette Smoke Exposure

**DOI:** 10.3389/fmolb.2021.663987

**Published:** 2021-04-26

**Authors:** Zhigang Wang, Wenzhang Liang, Cuiqing Ma, Jiachao Wang, Xue Gao, Lin Wei

**Affiliations:** ^1^Department of Immunology, Key Laboratory of Immune Mechanism and Intervention on Serious Disease in Hebei Province, Hebei Medical University, Shijiazhuang, China; ^2^Department of Intensive Care Unit, Hebei General Hospital, Shijiazhuang, China

**Keywords:** COPD, cigarette smoking, macrophage, bronchial epithelial cell, ciliary protein

## Abstract

Chronic obstructive pulmonary disease (COPD) is a chronic respiratory disease with high morbidity and mortality worldwide. So far, smoking is still its leading cause. The characteristics of COPD are emphysema and airway remodeling, as well as chronic inflammation, which were predominated by macrophages. Some studies have reported that macrophages were involved in emphysema and chronic inflammation, but whether there is a link between airway remodeling and macrophages remains unclear. In this study, we found that both acute and chronic cigarette smoke exposure led to an increase of macrophages in the lung and a decrease of ciliated cells in the airway epithelium of a mouse model. The results of *in vitro* experiments showed that the ciliary protein (β-tubulin-IV) levels of BEAS-2B cells could be inhibited when co-cultured with human macrophage line THP-1, and the inhibitory effect was augmented with the stimulation of cigarette smoke extract (CSE). Based on the results of transcriptome sequencing, we focused on the protein, bone morphogenetic protein-2 (BMP-2), secreted by the macrophage, which might mediate this inhibitory effect. Further studies confirmed that BMP-2 protein inhibited β-tubulin-IV protein levels of BEAS-2B cells under the stimulation of CSE. Coincidentally, this inhibitory effect could be nearly blocked by the BMP receptor inhibitor, LDN, or could be interfered with BMP-2 siRNA. This study suggests that activation and infiltration of macrophages in the lung induced by smoke exposure lead to a high expression of BMP-2, which in turn inhibits the ciliary protein levels of the bronchial epithelial cells, contributing to the remodeling of airway epithelium, and aggravates the development of COPD.

## Introduction

Chronic obstructive pulmonary disease (COPD) is a chronic respiratory disease with high morbidity and mortality worldwide. The 2015 Global Burden of Disease (GBD) Study estimated the global morbidity of COPD to be about 174 million (GBD 2015 Disease and Injury Incidence and Prevalence Collaborators, 2016), and it has the third ranking mortality after ischemic heart disease and cerebrovascular disease, that is, about 3.2 million deaths per year in 2015 (GBD 2015 Mortality and Cause of Death Collaborators, 2016). COPD has imposed a heavy global burden and will continue to increase in the future because of the aging population and the persisting air pollution (Mathers and Loncar, 2006). Smoking is still the leading cause of COPD (Mannino and Buist, 2007), although the proportion of male smokers decreased by 28% and that of female smokers decreased by 29% from 1990 to 2015. The WHO data showed that there were still about 1.1 billion people with a smoking habit in 2015 (GBD 2015 Risk Factors Collaborators., 2016), and its consequence would show up in the following decades.

One characteristic of COPD is emphysema (Vogelmeier et al., 2017). Most of the previous studies have been focused on the pathogenesis of emphysema, including gene pre-condition, early life events (Martinez, 2016), the imbalance of proteolysis/anti-proteolysis (Agusti and Hogg, 2019) and oxidation/anti-oxidation, and an enhanced apoptosis (Tuder et al., 2003). Although the exact mechanism is still unclear, there is a consensus that unquenched chronic inflammation leads to the disease (Barnes, 2013, 2014). Inflammation is characterized by the infiltration of neutrophils, macrophages, and lymphocytes in the airways and lung parenchyma (Hogg et al., 2004; Brusselle et al., 2011; Faner et al., 2013). These inflammatory cells secrete a variety of proteinases, such as, neutrophils elastase (Hunninghake and Crystal, 1983; Ghosh et al., 2019), granulase (Ngan et al., 2009; Kim et al., 2013), matrix metalloproteinase (Ghosh et al., 2019; Jeon et al., 2019), and perforin (Morissette et al., 2007; Zhang et al., 2014), which break down the extracellular matrix, such as collagen and elastin, causing structural damage to the alveoli and small airways, thus leading to emphysema.

Another consequence of COPD is remodeling of the airway wall (Hogg et al., 2004), showing squamous metaplasia, hypertrophy of submucosal glands, hyperplasia of smooth muscle, and fibrosis of adventitia in the airway. Normal airway epithelium consists of basal cells, ciliated cells, secretory cells (goblet cells, plasma cells, and Clara cells), neuroendocrine cells, and a few unclassified or intermediate cells (Mercer et al., 1994). Among them, ciliated cells are the main cell type (accounting for 50–70%) in the human airway (Boers et al., 1998; Montoro et al., 2018). They play a central role in the mucociliary clearance (MCC) function of the lung to get rid of the inhaled xenobiotics. Decreased ciliated cells, shorter cilia, and uncoordinated cilia beating frequency take a large part in the impaired MCC of patients with COPD (Randell, 2006; Rock et al., 2010).

Emphysema and airway remodeling of COPD are caused not only by cigarette smoking itself but also by the inflammation involved. Macrophages are the most prominent inflammatory cells in patients with COPD. Most previous studies reported that macrophage was related to emphysema (Morris et al., 2003; Hume et al., 2020; Xia et al., 2021), while few groups have reported that it was involved in airway remodeling (Ferhani et al., 2010; Bu et al., 2020), and its effect on cilia remains unclear. This study focused on the effect of macrophages on cilia in the airway epithelium of mice and ciliary protein levels of bronchial epithelial cells after exposure to cigarette smoke (CS) and its possible mechanism.

We used a whole-body CS exposure to induce COPD in mice. *In vivo*, we found that both acute and chronic CS exposure in mice could lead to an increase of macrophages in the lung and a decrease of ciliated cells in the airway epithelium. An *in vitro* study showed that THP-1 cells could inhibit β-tubulin-IV levels of BEAS-2B cells under the stimulation of CS extract (CSE). Further study confirmed that bone morphogenetic protein-2 (BMP-2) secreted by macrophages was responsible for that inhibitory effect.

## Materials and Methods

### Mice and CS Exposure

All C57BL/6N mice (6-week-old male) were purchased from Beijing Weitonglihua Laboratory Animal Technology Co., Ltd. The number of the Laboratory Animal Quality Certificate is 11400700223102. All experimental procedures were performed in compliance with the Institutional Animal Welfare Guidelines and were carried out according to the criteria outlined in the Guide for the Care and Use of Laboratory Animals [National Research Council (US) Committee for the Update of the Guide for the Care, and Use of Laboratory Animals, 2011] and with the approval of the Animal Care and Use Committee of Hebei Medical University. The mice were maintained in an animal facility under a 12-h light/dark cycle and were fed standard chow and sterile tap water. After raising them for 2 weeks for adaptation, the mice were divided into control (normal group) and experimental (CS group) groups. The experimental groups were exposed to Hongmei brand CS (tar oil 15 mg, nicotine 1.2 mg, produced by Yunnan Kunming Cigarette Factory, China). The cigarettes were burned in the combustion chamber, and then CS and fresh air were blown into the exposure chamber with a flow rate of 1:2. Acute CS exposure model lighting up two cigarettes every 20 min for five times every morning and every afternoon, respectively, with 6-h intervals, 5 days a week for 4 weeks. Chronic CS exposure model is a similar method to the acute exposure model: light up one cigarette every 20 min and the exposure time lasted for 16 weeks.

### Specimen Tissue Acquisition

Mice were anesthetized by intraperitoneal injection of ketamine (90 mg/kg) and xylazine (9 mg/kg) and were fixed on the animal operating table. The chest and the left ventricle were cut open, and blood was let out. In order to estimate the blood cells in the pulmonary vascular system, the lung was flushed by saline through the pulmonary artery until it became white. The airway and the lung were carefully separated, and then, they were processed differently. Some of the lungs were inflated and fixed for 6 h through intratracheal instillation of 4% paraformaldehyde under 20 cm H_2_O pressure. The trachea and the lung were separately embedded with paraffin and then sectioned. The rest of the fresh lungs were ground gently, sieved with a 70-um nylon BD Falcon cell strainer (BD Biosciences, San Jose, CA, United States), washed with sterile phosphate-buffered saline (PBS) (Gibco-BRL, Gaithersburg, MD, United States) without Ca^2+^ and Mg^2+^ supplemented with 2 mM EDTA (Sigma-Aldrich, St. louis, MO, United States), and then suspended in RPMI 1640 culture medium to form single-cell suspension. The rest fresh tracheae were put into liquid nitrogen, and then they were ground to extract the tissue RNA and protein.

### Fluorescence-Activated Cell Sorting (FACS)

The single-cell suspensions were labeled with 5 μl of PE anti-mouse F4/80 and 1.25 μl of APC anti-mouse/human CD11b as markers for macrophages or 5 μl of PE Rat IgG2a and 1.25 μl of APC Rat IgG2b as markers for control for 45 min in PBS on ice. All these antibodies were purchased from BioLegend, San Diego, CA, United States. Propidium iodide was added to exclude dead cells, and FACS was performed in the BD flow cytometry facility; 3 × 104 events were recorded.

### Reverse Transcription PCR (RT-PCR)

RNA was extracted with the RNeasy micro kit (QIANGEN, Beijing, China), and cDNA was synthesized with PrimeScript IV 1st strand cDNA Synthesis Mix (Tarkara, Kusatsu, Japan). PCR was performed with SYBR green chemistry in a Step One Plus (Applied Biosystems, Grand Island, NY, United States), and data were analyzed using the 2^–ΔΔCt^ method. The primers shown in Table 1 were synthesized by Invitrogen.

**TABLE 1:** Primers used for RT-qPCR.

Primer	Sequence
ACTB sense	5′-GTTG GTTG GAGC AAAC ATCC C-3′
ACTB antisense	5′-TTAG GAGT GGGG GTGG CTTT-3′
Foxj1 sense	5′-GGGT CGCA GAAT GGAA GTGA-3′
Foxj1 antisense	5′-GAGC CTTG GCGT TGAG AATG-3′
GAPDH sense	5′-CCTC TGAC TTCA ACAG CGAC AC-3′
GAPDH antisense	5′-CACC ACCC TGTT GCTG TAGC CA-3′
BMP-2 sense	5′-CTGC GGTC TCCT AAAG GTCG-3′
BMP-2 antisense	5′-GGGG TGGG TCTC TGTT TGAG-3′

### Western Blot

For airway analysis, the tracheae of the mice were homogenized in RIPA buffer (1% NP-40, 0.5% sodium deoxycholate, 0.1% SDS in PBS) containing phosphatase inhibitor cocktail (Sigma-Aldrich, St. louis, MO, United States). BEAS-2B cells were collected in cell lysis buffer containing 20 mM Tris–HCl, 150 mM NaCl, 1 mM EDTA, 1 mM EGTA, 1% Triton-X 100, 1.0 μg/ml leupeptin, 10 μg/ml aprotinin, 0.2 mM phenylmethylsulfonyl fluoride, 1 mM sodium orthovanadate, 0.1 mM sodium fluoride, 2.5 mM sodium pyrophosphate, and 1 mM β-glycerophosphate. Tissue lysates or cell lysates were centrifuged, and supernatant proteins were separated on 10% gradient SDS-PAGE and transferred to PVDF membranes (Millipore, Billerica, MA, United States). The membranes were blotted against antibodies to β-tubulin-IV (Abcam, Cambridge, MA, United States), and β-actin (Cell Signaling Technology, Danvers, MA, United States) or GAPDH (Cell Signaling Technology, Danvers, MA, United States). Primary antibody binding was detected with secondary antibodies conjugated with horseradish peroxidase and enhanced chemiluminescence (Amersham Pharmacia Biotech, Amersham, United Kingdom).

### Immunohistochemistry

Sections were deparaffinized, rehydrated, and subjected to antigen retrieval by autoclaving (10 min, 120°C, 30 psi) for 10 min in the citrate target retrieval solution. Subsequently, endogenous peroxidase was quenched with 3% H_2_O_2_ and blocked for 20 min with 10% goat serum. Primary rabbit anti-mouse CD68 polyclonal antibody (ABclonal, Wuhan, China) was added overnight at 4°C in 10% BSA-PBS (1:200). Sections were washed with PBS and then incubated with a biotinylated goat anti-rabbit secondary antibody (1:100) for 60 min followed by a 15-min treatment with streptavidin-horseradish peroxidase (Dako, Glostrup, Denmark). The antigen of interest was visualized using the brown chromogen 3,3-diaminobenzidine (Dako, Glostrup, Denmark) and counterstained with Harris’ hematoxylin solution (Sigma-Aldrich, St. louis, MO, United States). Sections were then dehydrated and mounted with Cytoseal 60 (Richard-Allan Scientific, Kalamazoo, MI, United States). Antibody dilutions and all washes were immersed in Tris-buffered saline solution. The section was scanned by TissueFAXS and analyzed by TissueFAXS Cytometry.

### Immunofluorescent Staining

Sections were deparaffinized, rehydrated, and subjected to antigen retrieval by autoclaving (10 min, 120°C, 30 psi) for 10 min in the citrate target retrieval solution. Primary rabbit anti-β-tubulin-IV antibody (Abcam) was added overnight at 4°C in 10% BSA-PBS (1:100). Sections were washed with PBS and then were incubated in dark with FITC-labeled goat anti-rabbit IgG (KPL, Gaithersburg, MD, United States) for 2 h at room temperature. Sections were washed three times in dark with PBS and sealed with ProLong Gold Antifade Reagent with DAPI (Cell Signaling Technology, Danvers, MA, United States). The section was scanned by Tissue FAXS and analyzed with StrataQuest (Tissue Gnostics GmbH, Vienna, Austria) and Tissue quest software.

### Cigarette Smoke Extract Preparation

Cigarette smoke extract was prepared by bubbling the CS from one commercially available cigarette (Hongmei, China; tar oil 15 mg, nicotine 1.2 mg) into 4 mL of RPMI 1640 medium (Life Technologies, Carlsbad, CA, United States), containing no serum or growth factors, using a modified method described previously. This was considered as a 100% CSE solution and then sterilized and stored at −80°C (Xu et al., 2012; Kratzer et al., 2013a; Sakhatskyy et al., 2014).

### Cell Lines, Culture Media, and Growth Conditions

The BEAS-2B, simian virus 40-transformed, immortalized bronchial epithelial cell line used for this study (Reddel et al., 1988) was preserved in our laboratory. This cell line has been cultured continuously for >100 passages. In this study, passages 55 and 65 were used. The cells were cultured in extracellular matrix (ECM)-coated dishes in RPMI 1640 medium supplemented with 10% heat-inactivated-FCS, 100 U/mL penicillin, and 100 mg/mL streptomycin (Gibco, Carlsbad, CA, United States) at 37°C in a humidified atmosphere of 5% CO_2_ in air. ECM-coated dishes were prepared by incubating wells with 1 ml of 50 ug/ml of collagen IV (Sigma, St. louis, MO, United States) per well overnight and washed with sterile PBS before cells were seeded; 1 × 103 cells were seeded in each well overnight, then cultured with the stimulation of CSE and/or recombinant BMP2 protein (PeproTech, Rocky Hill, NJ, United States) for 48 h or pre-treated with LDN193189 (MCE, Monmouth, NJ, United States) for 24 h, and then co-cultured with 5 × 105 THP-1 cells in Transwell with the stimulation of CSE for 48 h.

Human monocytic leukemia cell line THP-1 (American Type Culture Collection, Manassas, VA, United States) cells were maintained in RPMI 1640 media with the addition of 10% (v/v) heat-inactivated-FBS at 37°C in a 5% CO_2_ humidified atmosphere. Cells were seeded at 5 × 105 cells per well on Transwell filters and treated with phorbol myristate acetate (PMA 30 ng/ml, Multi Sciences, China) for 24 h to induce differentiation and filter attachment, and co-cultured with Beas-2B cells or pre-incubated with siRNA for 24 h, and then co-cultured with Beas-2B cells with the stimulation of CSE.

### Co-culture

BEAS-2B cells were seeded at 1 × 103 cells in 6-well cell culture plates for 24 h. At the same time, THP-1 cells were seeded at 5 × 105 cells per well on Transwell filters and treated with PMA for 24 h. The next day, the Transwell inserts were placed in the 6-well cell culture plate and were stimulated with CSE for 48 h before harvesting.

### RNA-Seq Analysis

Total RNA was isolated from THP-1 cells in three different conditions, including THP-1 cells activated by PMA (group 1), THP-1 cells co-cultured with BEAS-2B cells (group 2), and THP-1 cells co-cultured with BEAS-2B cells and stimulated by CSE (group 3). Construction of the cDNA library and sequencing were performed by Sinotech Genomics, ShanHai, China using the Illumina Novaseq 6000 sequencing platform. High-quality reads were mapped with Homo sapiens GRCh38, using Hisat2 version 2.0.4. The expression level of each gene was standardized to fragments per kilobase of exon model per million mapped reads (FPKM) using StringTie version 1.3.0 and trimmed mean of *M* values (TMM) (*Q* value < 0.05, fold change ≥2 times).

### SiRNA

THP-1 cells were seeded at 5 × 105 cells per well on Transwell filters and treated with PMA for 24 h; siBMP2 was transfected into cells with Lipofectamine 2000 (Invitrogen, Carlsbad, CA, United States) following the instructions of the manufacturer for 24 h and co-cultured with Beas-2B cells for 48 h with the stimulation of CSE. The sequence of siBMP2 (Ribobiotech, GuangZhou, China) was listed as:

sense: 5′UCAACUCUGUUAACUCUAA3′
antisense: 5′UUAGAGUUAACAGAGUUGA3′

### Statistical Analysis

SPSS statistical software (version 16.0) was used for statistical analysis. Data were expressed as mean plus SD. The significance between the two groups is determined using *t*-test. ^∗^*P* < 0.05 was considered statistically significant. All experiments were performed at least three times.

## Results

### Acute CS Exposure Led to an Increase of Macrophages in the Lung and a Decrease of β-Tubulin-IV Levels in the Airway

**FIGURE 1:**
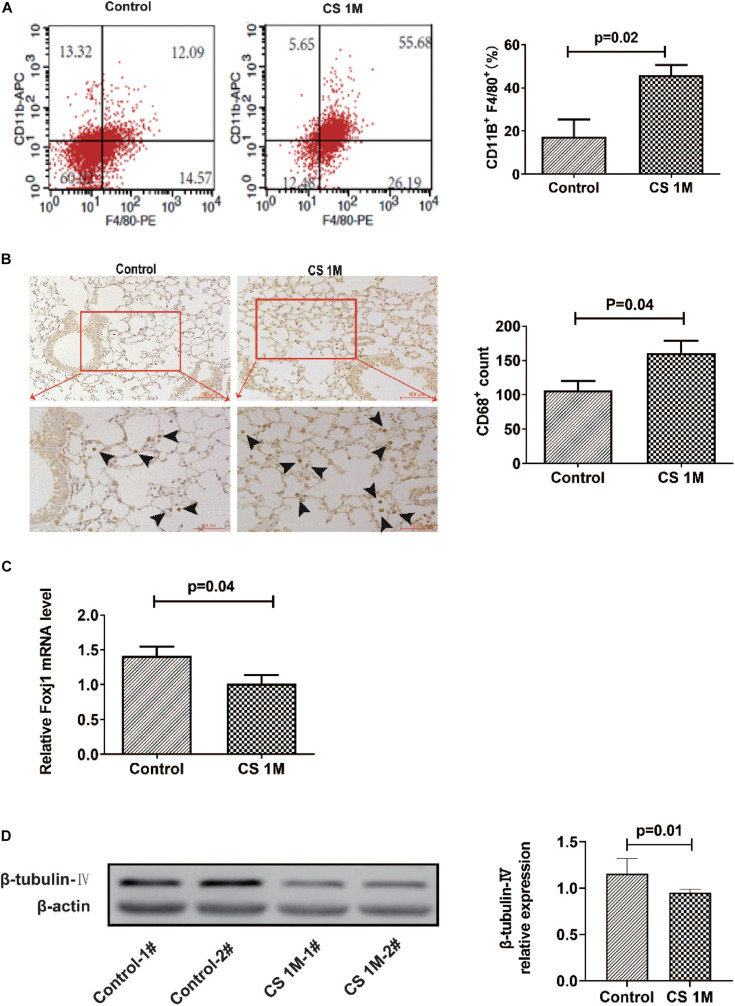
The changes of macrophages in the lung and β-tubulin-IV in the airway of mice after acute cigarette smoke (CS) exposure for 4 weeks. **(A)** The percentage of CD11b^+^F4/80^+^ macrophages in the lung detected by fluorescence-activated cell sorting (FACS) in the control group and the CS1M group (*n* = 4, CS1M = cigarette smoke exposure for 4 weeks). **(B)** The CD68^+^ macrophages in the lung detected by immunohistochemical (IHC) staining in the control group and the CS1M group (the up panel bar is 100 μm, the bottom panel bar is 50 μm, black arrow indicates CD68^+^ macrophages, *n* = 6). **(C)** The relative expression of foxj1 mRNA in the airway of mice was determined by qRT-PCR after acute CS exposure for 4 weeks (*n* = 6). **(D)** The relative levels of β-tubulin-IV protein in the airway of mice were quantitated by densitometry and normalized to β-actin after acute CS exposure for 4 weeks (*n* = 6).

In our experiment, we first explored the effect of acute high-dose CS exposure on macrophages in the lung and ciliary protein levels in the airway of mice. We took CS exposure (20 cigarettes per day) for 4 weeks as the acute exposure condition because the restoration of epithelial histology is about 2 weeks after its extensive damage caused by inhalation of SO_2_ in mice (Rawlins and Hogan, 2008). After acute CS exposure, mice were anesthetized and sacrificed, the lungs were freshly made into single-cell suspension for FACS or were fixed and sectioned for immunohistochemistry (IHC), and the airways were homogenized to extract RNA and protein for RT-PCT and Western blot. The results showed that the percentage of CD11b^+^F4/80^+^ macrophages detected by FACS increased significantly in the CS group (45.89 ± 4.73%) compared to the control group (17.43 ± 7.90%) (Figure 1A). Also, the number of CD68^+^ macrophages in the lung section detected by IHC staining also increased evidently from 106.3 per 10 random HPFs (high power fields) in the control group to 160.2 in the CS group (Figure 1B). After acute CS exposure, the expression of both foxj1 mRNA and β-tubulin-IV protein of the airway decreased significantly in the CS group compared to that of the control group (Figures 1C,D).

### Chronic CS Exposure Resulted in the Development of Emphysema and Remodeling of the Airway Wall in Mice

**FIGURE 2:**
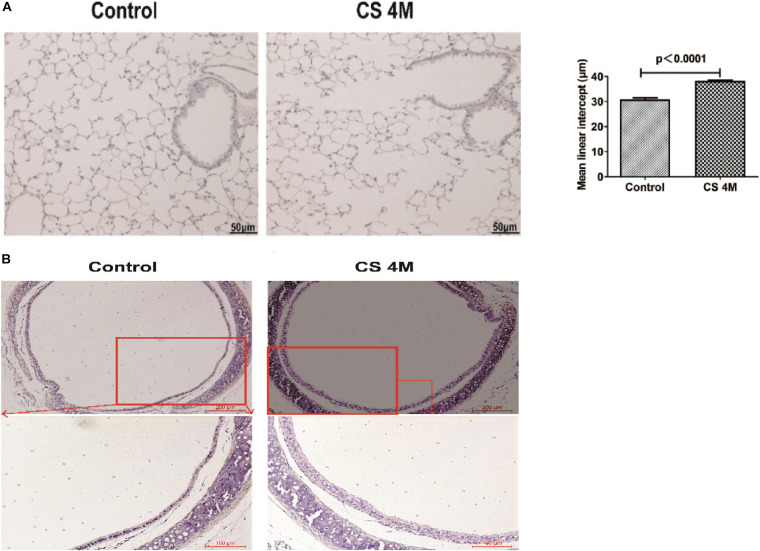
The H&E staining of the airway and lung section of mice after chronic CS exposure for 16 weeks. **(A)** H&E staining of the lung section of mice in the control group and the CS4M group after chronic CS exposure for 16 weeks (*n* = 4, bar = 50 μm, CS4M = cigarette smoke exposure for 16 weeks). Mean linear intercept of alveoli of mice in the control group and the CS4M group after chronic cigarette smoke exposure for 16 weeks. **(B)** H&E staining of the airway section of mice in the control group and the CS4M group after chronic CS exposure for 16 weeks (*n* = 4, the up panel bar is 200 μm, the bottom panel bar is 100 μm).

According to the commonly used methods in a previous study, animal models usually took 4–6 months to display signs of disease (Ofulue and Ko, 1999; Kratzer et al., 2013b). Different from the acute exposure condition, mice were exposed to CS (10 cigarettes per day) for 16 weeks to induce emphysema in chronic exposure mode and were successfully induced for the development of emphysema in the CS group. H&E staining of lung sections showed that, in the CS group, most of the integrity of small airways was destroyed, the alveolar structure was seriously damaged, alveolar fusion was evident, and the mean alveolar intercept was 38.20 ± 0.40 μm, which was significantly enlarged compared to that of the control group, indicating that emphysema was successfully induced by long-term CS exposure. While, in the control group, small airway integrity was retained and just a small amount of alveolar destruction was observed, alveolar fusion was mild, and the mean alveolar intercept was 30.34 ± 0.44 μm (Figure 2A). As we know, apart from emphysema, the other characteristic of COPD is remodeling of the airway wall. Then, we examined the airway histological profile of mice. The results showed that the airway epithelium of the CS group was thicker than that of the control group, the cell layers were increased by three to four layers, and its arrangement was disordered in the CS group, while it was one or two aligned cell layers in the control group (Figure 2B). These epithelial histological changes indicated that there was the remodeling of the airway wall after chronic CS exposure for 16 weeks.

### Chronic CS Exposure Led to an Increase of Macrophages in the Lung and a Decrease of Ciliated Cells in the Airway of Mice

**FIGURE 3:**
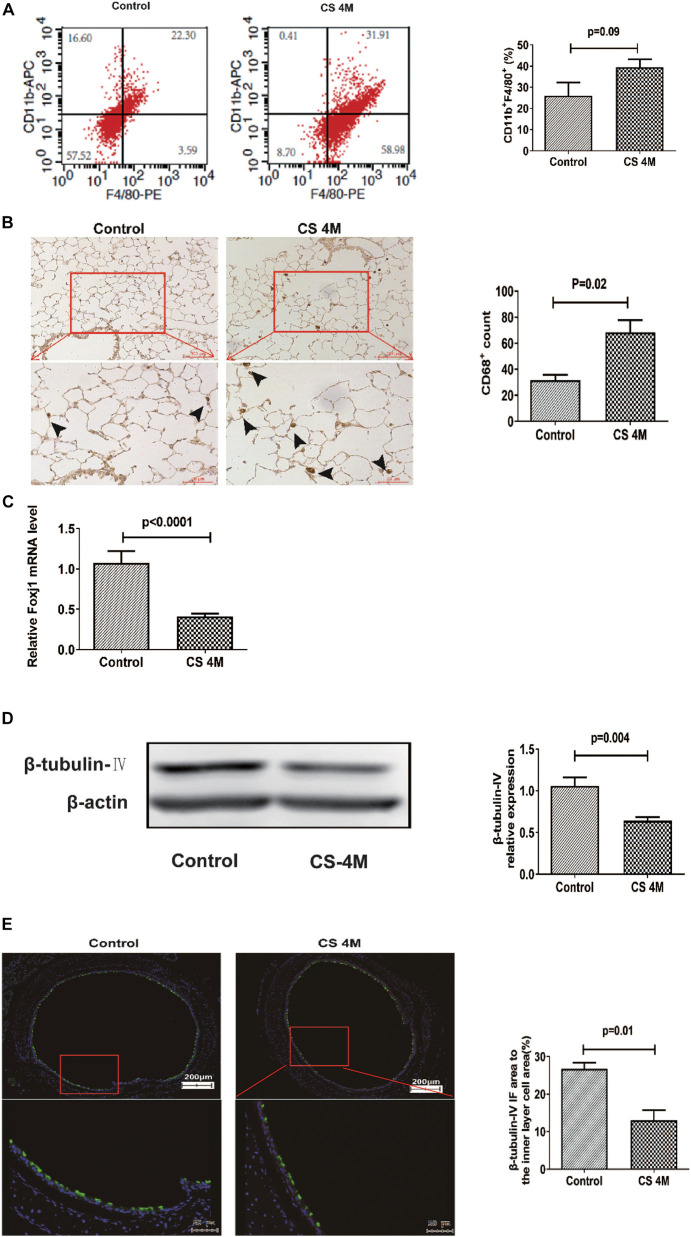
The changes of macrophages in the lung and β-tubulin-IV in the airway of mice after chronic CS exposure for 16 weeks. **(A)** The percentage of CD11b^+^F4/80^+^ macrophages in the lung detected by FACS in the control group and the CS4M group (*n* = 4, CS4M = cigarette smoke exposure for 16 weeks). **(B)** The CD68^+^ macrophages in the lung detected by IHC staining in the control group and the CS4M group (the upper panel bar is 100 μm, the bottom panel bar is 50 μm, black arrow indicates CD68^+^ macrophages, *n* = 6). **(C)** The relative expression of foxj1 mRNA in the airway of mice was determined by qRT-PCR after chronic CS exposure for 16 weeks (*n* = 6). **(D)** The relative levels of β-tubulin-IV protein in the airway of mice were quantitated by densitometry and normalized to β-action after chronic CS exposure for 16 weeks (*n* = 6). **(E)** The β-tubulin-IV protein levels in the airway epithelium of mice stained by IF in the control group and the CS4M group (the upper panel bar is 200 μm, the bottom panel bar is 50 μm, nuclear was dyed with DAPI, and cilia was dyed with FITC, *n* = 4). The ratio of cilia area to the inner layer cell area in the control group and the CS4M group.

In this study, after chronic CS exposure for 16 weeks in the mouse model, macrophages increased in the CS group compared to the control group, but there was a little difference between different marker performances. The percentage of CD11b^+^F4/80^+^ macrophages in the lung detected by FACS was 39.49 ± 3.70% in the CS group and 26.01 ± 6.22% in the control group. Although the number of macrophages in the CS group increased, there was no significant difference between the two groups (Figure 3A). However, the number of macrophages detected by IHC was significantly different between the two groups, from 31.67 ± 4.05 per 10 random HPFs in the control group to 68.50 ± 9.37 in the CS group (Figure 3B).

Although there was a slight difference in the macrophage performance, it was consistent with the change of ciliated cells in the airways after acute and chronic CS exposure. After chronic CS exposure, the expression of foxj1 mRNA detected by RT-PCR (Figure 3C) and β-tubulin-IV protein detected by Western blot (Figure 3D) in the airways both decreased significantly in the CS group compared with that in the control group. We also detected β-tubulin-IV protein of cilia in the epithelium by IF, and the images showed that the cilia dyed with green color were interspersed in the inner layer of the airway, its distribution was non-uniform, and the intensity was weakened or even lost in some area in the CS group, while it was uniform in the control group. The ratio of cilia area to the inner layer cell area was 15.6% in the CS group and 23.4% in the control group, and there was a significant difference between the two groups (Figure 3E). These results indicated that CS exposure led to a decrease of ciliated cells.

### THP-1 Cells Inhibited β-Tubulin-IV Levels of BEAS-2B Cells Under the Stimulation of CSE

**FIGURE 4:**
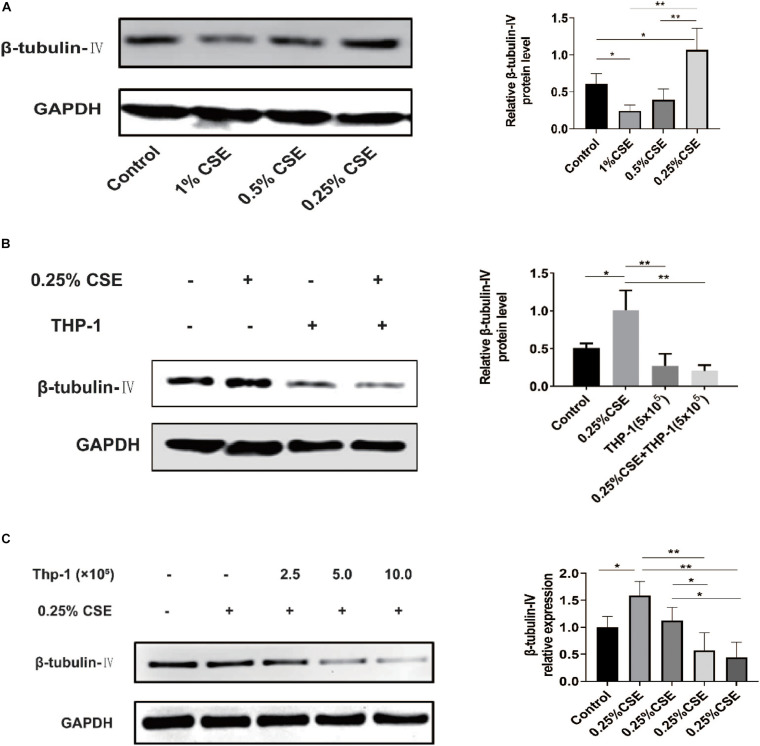
β-tubulin-IV protein levels of BEAS-2B cells after the stimulation of cigarette smoke extract (CSE) and/or THP-1 cells. **(A)** β-tubulin-IV protein levels of BEAS-2B cells after the stimulation of different concentrations of CSE. **(B)** β-tubulin-IV protein levels of BEAS-2B cells when co-cultured with THP-1 cells under the stimulation of 0.25% CSE. **(C)** β-tubulin-IV protein levels of BEAS-2B cells when co-cultured with different numbers of THP-1 cells under the stimulation of 0.25% CSE. All β-tubulin-IV protein levels were quantitated by densitometry and normalized to GAPDH (*P < 0.05, **P < 0.01).

*In vivo*, we confirmed that acute and chronic CS exposure both lead to macrophage infiltration and ciliated cell reduction. *In vitro*, we tried to explore whether there is some relationship between macrophages and ciliated cells. First, we tested the effect of CSE on β-tubulin-IV levels of bronchial epithelial cells. The results showed that the levels of β-tubulin-IV of BEAS-2B cells decreased with the stimulation of 1% CSE for 48 h, while they increased with 0.25% CSE stimulation for 48 h (Figure 4A). Next, we studied the effect of macrophages on β-tubulin-IV levels of bronchial epithelial cells. The results showed that THP-1 cells could inhibit β-tubulin-IV levels of BEAS-2B cells, and then when 0.25% CSE was added into the medium, the inhibitory effect was augmented (Figure 4B). Furthermore, this inhibitory effect was further enhanced with increased numbers of THP-1 cells from 2.5 × 10^5^ to 10 × 10^5^ (Figure 4C).

### Bone Morphogenetic Protein-2 Was Screened Out and Verified to Have an Inhibitory Effect on β-Tubulin-IV Levels

**FIGURE 5:**
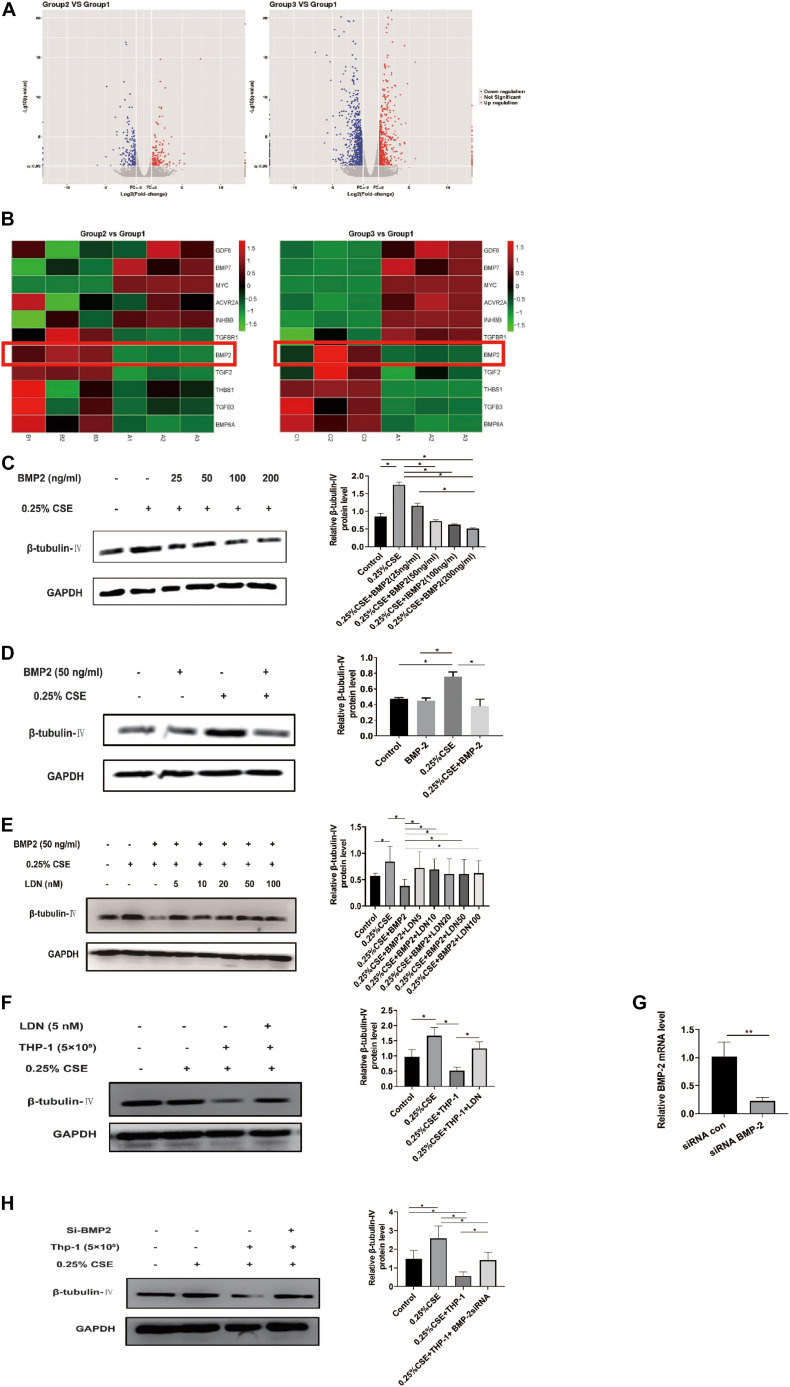
Bone morphogenetic protein-2 (BMP-2) protein has an inhibitory effect on β-tubulin-IV levels of Beas-2B cells. **(A)** The volcano images of genes whose expression difference was more than two-fold in THP-1 cells co-cultured with BEAS-2B cells (group 2, **B**) vs. THP-1 cells (group 1, **A**) and THP-1 cells co-cultured with BEAS-2B cells under stimulation of CSE (group 3, **C**) vs. THP-1 cells (group 1, **A**). **(B)** The heat map of genes related to TGF-β superfamily in group 2 vs. group 1 and group 3 vs. group 1. **(C)** β-tubulin-IV levels of BEAS-2B after being stimulated with 0.25% CSE and different concentrations of recombinant human BMP-2 protein. **(D)** β-tubulin-IV levels of BEAS-2B after being stimulated with 0.25%CSE and 50 ng/ml recombinant human BMP-2 protein. **(E)** β-tubulin-IV levels of BEAS-2B pre-treated with different concentrations of BMP receptor inhibitor LDN before being stimulated with 0.25%CSE and 50 ng/ml recombinant human BMP-2 protein. **(F)** β-tubulin-IV levels of BEAS-2B pre-treated with 5 nM LDN before co-cultured with THP-1 cells and being stimulated with 0.25% CSE. **(G)** BMP2 mRNA expression of THP-1 cells pre-incubated with siBMP2 for 24 h. **(H)** β-tubulin-IV levels of BEAS-2B after being stimulated with 0.25% CSE and co-cultured with THP-1 cells pre-incubated with siBMP2. All β-tubulin-IV protein levels were quantitated by densitometry and normalized to GAPDH (*P < 0.05, **P < 0.01). Red box indicates the result of BMP-2 in heatmap of RNA-seq analysis between groups.

Now that THP-1 cells could inhibit β-tubulin-IV levels of BEAS-2B cells when they were co-cultured in Transwell without direct contact, this inhibitory effect might come from certain soluble factors secreted by activated THP-1 cells. Transcriptome array technology was used to test RNA expression profiles of THP-1 cells with different conditions, namely, THP-1 cells (group 1), THP-1 cells co-cultured with BEAS-2B cells (group 2), and THP-1 cells co-cultured with BEAS-2B cells under the stimulation of CSE (group 3). Taking RNA differential expression of more than two-fold as standard, compared with group 1, the differentially expressed genes were 374 (172 upregulated and 202 downregulated) in group 2 and were 1,465 (594 upregulated and 871 downregulated) in group 3, which suggested that the effect of co-culture under the stimulation of CSE was far more complicated than that of co-culture alone (Figure 5A). We speculated that genes that have an inhibitory effect on the β-tubulin-IV protein levels of BEAS-2B should be soluble substances and should be upregulated in group 2 and group 3 at the same time. We noticed that many of these differentially expressed genes belonged to the TGF-β superfamily, which was associated with airway epithelial cell differentiation. Eventually, BMP-2 was screened out as the target (Figure 5B).

In order to verify if BMP-2 could affect the β-tubulin-IV levels, different concentrations (25, 50, 100, and 200 ng/ml) of recombinant human BMP-2 protein was added into the medium when BEAS-2B cells were cultured under the stimulation of 0.25% CSE. Results showed that the β-tubulin-IV levels were inhibited in a concentration-dependent manner from 25 to 200 ng/ml. When the concentration reached 50 ng/ml, β-tubulin-IV levels were inhibited down below the normal level (Figure 5C), indicating that BMP-2 could inhibit the β-tubulin-IV levels of BEAS-2B cells. In the following experiments, the concentration of recombinant human BMP-2 protein was fixed to 50 ng/ml, because at this point, the β-tubulin-IV levels of BEAS-2B was not affected without the stimulation of CSE (Figure 5D).

To further verify that BMP-2 could inhibit the β-tubulin-IV levels of BEAS-2B cells, we interfered with the BMP signal using BMP-2 blockage LDN or reducing BMP-2 production with BMP-2 siRNA in THP-1 cells.

First, BEAS-2B cells were pre-treated with BMP blockage LDN for 24 h before CSE and recombinant human BMP-2 stimulation; the inhibitory effect of BMP-2 on β-tubulin-IV levels of BEAS-2B cells could be completely blocked at a very lower dose of LDN (5 nM), which was similar to the other experimental dosages of LDN from 10 to 100 nM (Figure 5E).

Then, we tested whether LDN would block the inhibitory effect of THP-1 cells on β-tubulin-IV levels of BEAS-2B cells. The results showed that the β-tubulin-IV levels of BEAS-2B cells could be mostly restored when the BEAS-2B cells were pre-treated with LDN for 24 h before they were co-cultured with THP-1 cells and stimulated by CSE (Figure 5F).

Subsequently, we interfered THP-1 cells with BMP-2 siRNA, which turned out to decrease BMP-2 mRNA expression by over 70% (Figure 5G). The results showed that the inhibitory effect of THP-1 cells on β-tubulin-IV levels of BEAS-2B cells could also be mostly blocked when THP-1 cells interfered with BMP-2 siRNA for 24 h before they were co-cultured with BEAS-2B cells and stimulated by CSE (Figure 5H).

Both LDN and BMP-2 siRNA mostly block the inhibitory effect of THP-1 cells on the β-tubulin-IV levels of BEAS-2B cells, demonstrating that BMP-2 produced by THP-1 cells plays an essential role in inhibiting the β-tubulin-IV levels of BEAS-2B cells.

## Discussion

Airway epithelium, which plays a key role in the mucosal defense response of the host to pathogens, consists of about 50–70% ciliated cells, 30% basal cells, secretory cells (up to 25% goblet cells, 11% Clara cells and some serous cells), neuroendocrine cells, and a few unclassified or intermediate cells (Mercer et al., 1994; Boers et al., 1998; Montoro et al., 2018). It is clear that ciliated cells are the main cell types of human airways. Each ciliated cell has about 300 motile cilia. Normal amounts of cilia and coordinated cilia beating frequency are necessary for normal mucociliary clearance (Wanner et al., 1996). Goblet cells mainly secrete mucin (Roy et al., 2014), which is coated on the surface of the airway, and capture inhaled particles. Then, these exotic particles can be expelled out of the airway through the beating of cilia. Clara cells secrete a 10 kD protein, called CC10 or CCSP, which is an anti-inflammatory and immunoregulatory protein (Chen et al., 2001; Mandal et al., 2004; Liu et al., 2013). Normal cilium structure and function, the appropriate amount of mucus as well as physicochemical properties, and the appropriate amount of lining fluid layer around the cilia constitute the normal MCC function. Impaired MCC function means that the ability to get rid of pathogens and other exotics is weakened. Apart from viral or bacterial pathogens, cigarette smoking has a profound impact on health.

Chemical analysis has identified more than 3,800 compounds in CS, which contain many harmful substances (Lofroth, 1989; Brunnemann and Hoffmann, 1991; Pryor and Stone, 1993; Hasday et al., 1999). Long-term chronic cigarette smoking exposure is known to cause the development of COPD, which has some conventional characteristics, such as emphysema and remodeling of the airway wall.

Cigarette smoke exposure is the most appropriate model to study COPD in mice, and several exposure methods are available (Tanner and Single, 2019). The protocols for CS exposure in mice vary greatly in length, frequency, and numbers of CS exposures, as well as in the exposure mode and cigarette being used (Fricker et al., 2014). Although these different methods of smoke generation affect smoke components and its concentrations, they seemingly do not significantly influence the animal disease state (Leberl et al., 2013). Differences of interspecies in animal models influence the development time of emphysematous phenotypes following CS exposure. Generally, it takes 4–6 months to display signs of disease in most animal models (Ofulue and Ko, 1999; Kratzer et al., 2013b). In this study, we used a whole-body exposure for 4 and 16 weeks, respectively, to establish mouse models of acute and chronic respiratory diseases.

Long-term chronic CS exposure causes significant changes in the airway epithelium. A link between smoking and decreased ciliated cells had already been established 60 years ago, which showed that the more the number of cigarettes, the more is the area of cilia absence (Auerbach et al., 1961). Human airway biopsy specimens showed that cilia were approximately 13% to 15% shorter in smokers than in healthy people who had never smoked (Leopold et al., 2009). Long-term chronic CS exposure in DAB/2 and C57BL/6J mice led to an increase in the area without cilia, disordered arrangement, and shortening of cilia in the area with cilia (Bartalesi et al., 2005). It is reported that CS exposure could affect the ciliogenesis by inhibiting the essential genes for ciliogenesis, such as MCIDAS and FOXJ1 (You et al., 2004; Tamashiro et al., 2009; Stubbs et al., 2012; Didon et al., 2013; Brekman et al., 2014). Their downstream protein, acetylated α-tubulin (Griggs et al., 2017) or β-tubulin-IV (Li et al., 2017), is often used as a specific symbol of ciliated cells. Some research showed that when primary human bronchial epithelial cells were cultured at air-liquid interface, CS exposure could decrease the number of ciliated cells and acetylated tubulin protein levels (Schamberger et al., 2015), while roflumilast N-oxide could resume β-tubulin-IV protein levels (Milara et al., 2012). In this experiment, both acute and chronic CS exposure in mice could lead to decreased expression of foxj1 mRNA and of β-tubulin-IV protein in the airway. In chronic CS exposure mode, we also detected β-tubulin-IV protein in the airway through immunofluorescence. Because the immunofluorescence staining was continuous, it was hard to tell which cilia came from which cell, so it was impossible to distinguish β-tubulin-IV-positive cells from negative ones. We had to quantify the ratio of the immunofluorescence staining area to the inner layer cells area. The results showed that this proportion was 15.6% in the CS group and 23.4% in the control group, verifying that CS exposure led to a decrease of ciliated cells. Compared with the data from previous studies that ciliated cells account for more than half of the epithelial cells, the proportion of ciliated cells in this study was reduced. There might be two reasons: One reason is that the ratio of the fluorescence area to the inner layer cells area itself leads to a decrease in the proportion of ciliated cells; the other reason might be due to the deviation of immunofluorescence staining of paraffin sections. Anyway, the decrease of ciliated cells caused by cigarette smoking is consistent.

Patients with COPD experience significant changes in the airway epithelium that not only impede pathogen clearance but also trigger an inflammatory response (Rock and Hogan, 2011). This inflammation is evident throughout the tracheal tree and lung tissue, presenting with increased infiltration of neutrophils, macrophages, and lymphocytes (Hogg et al., 2004; Brusselle et al., 2011; Faner et al., 2013). Macrophages and neutrophils could release proteases, namely, neutral elastin, MMP, and cathepsin, by degrading the ECM, leading to lung tissue damage (Chapman and Shi, 2000). Lymphocyte infiltration was prominent with CD8^+^T cells (Saetta et al., 1999; Caramori et al., 2016). CD8^+^T cells secrete various soluble cytokines and chemokines to recruit and activate macrophages producing more cytokines. The produced elastin fragments act as a monocyte chemokine to enhance macrophage-mediated lung injury (Small et al., 2001; Grumelli et al., 2004). Lymphocytes can also secrete macrophage migration inhibitors. Therefore, to some extent, macrophage aggregation depends on the presence of lymphocytes.

The results showed that, after acute CS exposure in mice, the number of macrophages in the lung of the CS group increased significantly compared with that of the control group detected by FACS and IHC, which was consistent with previous studies, suggesting that acute CS exposure could induce the accumulation of macrophages in the lung. After chronic CS exposure in mice, the number of CD68^+^ macrophages in the lung of the CS group increased significantly compared with that of the control group detected by IHC. But there was only an increasing trend of CD11b^+^F4/80^+^ macrophages in the lung of the CS group detected by FACS and without significant difference between the two groups. This mild difference might be caused by the specificity of different markers.

After chronic CS exposure, we successfully induced emphysema development in the mouse model. The pathogenesis of emphysema is complex and heterogeneous, that is, several mechanisms coexist and interact (Agusti and Faner, 2018), including, the imbalance of proteolytic/antiproteolytic enzyme and oxidation/anti-oxidation, increased epithelial cell apoptosis, and innate and adaptive immune abnormalities (Tuder and Petrache, 2012). CS exposure could induce emphysema in lac gene-deficient or SCID-deficient mice, indicating that the development of emphysema did not require adaptive immunity (D’hulst et al., 2005; De Cunto et al., 2016). However, other studies have shown that CD8^+^ T lymphocytes play a protective role in emphysema development induced by CS exposure (Maeno et al., 2007; Motz et al., 2010). This suggests that the role of adaptive immunity in COPD development remains controversial, but it is indisputable that innate immunity plays a role throughout the development of COPD. Increased alveolar macrophage numbers are clinically correlated with COPD severity (Di Stefano et al., 1998; Retamales et al., 2001). It has been suggested that persistent intrinsic immune activation contributes to the persistence of chronic airway inflammation. Airway epithelial progenitor cells provide upstream stimulation signals for chronic intrinsic immune activation, suggesting some interaction between undifferentiated airway epithelial cells and innate immunity, especially macrophages (Byers et al., 2013). How macrophages interact with airway epithelium is now a hot topic in the research.

We want to know if there is a relationship between macrophage and ciliary protein levels of airway epithelium.

The majority of previous literature reported that CS could lead to decreased ciliated cells (Milara et al., 2012; Schamberger et al., 2015; Amatngalim et al., 2018) or shorter cilia (Bartalesi et al., 2005). We first detected the impact of CSE on ciliary protein levels of BEAS-2B cells. Unexpectedly, the results showed that ciliary protein levels of BEAS-2B cells decreased with the stimulation of 1% CSE but increased with the stimulation of 0.25% CSE. This discrepancy may be due to fact that primary bronchial epithelial cells (PBECs) are far more sensitive to CSE stimulation than BEAS-2B cells, and even a lower concentration of CSE could result in decreased ciliated cells. Another possibility is that previous PBECs experiments did not study the effect of different concentrations of CSE on ciliated cells. It is due to the fact that mild exotic stimulation could increase ciliary protein levels or promote PBECs differentiated toward ciliated cells from a physiological protective perspective. In the following tests, we used 0.25% CSE as the stimulation condition.

When BEAS-2B cells and activated THP-1 cells were co-cultured in Transwell with or without the stimulation of CSE, the results showed that THP-1 cells could inhibit the ciliary protein levels of BEAS-2B cells. This inhibitory effect was significantly enhanced with CSE stimulation. Therefore, it is speculated that activated macrophages may affect the ciliation, which will be aggravated during cigarette smoking exposure in humans. Subsequently, we tested the transcriptome of three different kinds of THP-1 cells, namely, activated THP-1 cells by PMA, activated THP-1 cells co-cultured with BEAS-2B, and activated THP-1 cells co-cultured with BEAS-2B following the stimulation of CSE.

The number of differentially expressed genes was much higher when THP-1 and BEAS-2B cells were co-cultured under the stimulation of CSE than that of co-culture alone, which suggests that CSE causes a more complicated situation. Comparison among groups showed that there were 11 differentially expressed genes that belong to the TGF-β superfamily, which has more than 40 ligand members, involving in embryonic development, airway epithelial differentiation, tissue balance, and many disease states (Derynck and Zhang, 2003; Guo and Wang, 2009; Massague, 2012). We speculated that genes that have an inhibitory effect on β-tubulin-IV protein levels of BEAS-2B should be soluble substances and upregulated in group 2 and group 3 at the same time. Ruling out the other nine genes and TGIF2, which has a similar performance with BMP-2 but not stable between group 1 and group 3, BMP-2 protein was finally screened out as a probable target.

Bone morphogenetic protein signaling plays an essential role in the maintenance of mature lung tissue. In adult mice, defect in BMP/Smad signaling leads to abnormal pulmonary vascular remodeling and pulmonary hypertension (Huang et al., 2009), while the BMP-2 receptor inhibitor LDN could reverse squamous metaplasia and increase the number of ciliated cells and secretory cells (Lee et al., 2015). In primary bronchial epithelial cells from healthy volunteers and patients with cystic fibrosis, BMP activity inhibition could promote cell differentiation and increase the population of ciliated cells. Following stimulation with recombinant BMP, cell differentiation was blocked and ciliated cells decreased significantly (Cibois et al., 2015).

The results showed that, under the stimulation of CSE, recombinant human BMP-2 protein could decrease the ciliary protein levels of BEAS-2B cells in a dose-dependent manner. In order to further verify whether it was BMP-2 secreted by activated THP-1 cells that inhibits ciliary protein levels, we interfered with BMP signal either through blocking BMP signal transduction by BMP receptor blocker LDN or through decreasing the production of BMP-2 secreted from THP-1 cells by siRNA interference. The results showed that ciliary protein levels of BEAS-2B cells mostly reversed after LDN pre-treatment. Then, we used BMP-2 siRNA to interfere with THP-1 cells, and BMP-2 expression of THP-1 cells was inhibited by more than 70%, and ciliary protein levels of BEAS-2B cells were also restored. These results implied that the BMP-2 from activated macrophages will activate the BMP signal in bronchial epithelial cells, resulting in inhibition of ciliary protein levels.

In conclusion, enhancing BMP signal by exogenous recombinant human BMP-2 protein could inhibit the ciliary protein levels of BEAS-2B cells, and weakening BMP signal using BMP receptor blocker or decreasing the production of BMP-2 from THP-1 cells could reverse the inhibitory effect of THP-1 cells on the ciliary protein levels of BEAS-2B cells. This indicated that macrophages activated by CSE could secrete more BMP-2, which activates the BMP signal, leading to inhibition of ciliary protein levels.

What is the exact mechanism of BMP-2 inhibiting on the ciliary protein levels? It has been reported that, during the differentiation of neuroepithelial cells, BMP-2 could activate Smad1, which mediates Notch signal enhancement, inhibiting neuronal differentiation (Takizawa et al., 2003), suggesting that there is an interaction between BMP and Notch pathway, which is closely related to the differentiation of airway basal cell (Whitsett and Kalinichenko, 2011; Firth et al., 2014). Whether BMP-2 affects the ciliary protein levels through the Notch pathway will be further discussed in follow-up studies. Additionally, what is in the CSE mixture? Although we did not explore the exact substances that played an essential role in this study, generally, CSE compositions were as follows: 18.7% acetonitrile, 18.0% acetone, 12.5% 2-hydroxy-2-methyl-propanenitrile, 8.98% nicotine, and 5.86% nicotyrine (Kim et al., 2018). Without a doubt, knowing the exact composition of CSE will be helpful to explore the molecular mechanism of its effect on airway remodeling in future research.

## Data Availability Statement

The raw data supporting the conclusions of this article will be made available by the authors, without undue reservation.

## Ethics Statement

The animal study was reviewed and approved by the Animal Care and Use Committee of Hebei Medical University.

## Author Contributions

LW, ZW, and WL conceived and designed the research. ZW and WL performed the experiments, analyzed the data, and interpreted the results of the experiments. JW and XG prepared the figures and drafted the manuscript. LW and CM edited and revised the manuscript, and approved the final version of the manuscript. All authors contributed to the article and approved the submitted version.

## Conflict of Interest

The authors declare that the research was conducted in the absence of any commercial or financial relationships that could be construed as a potential conflict of interest.
